# “Digging Deeper” into the Relationship Between Safety Climate and Turnover Intention Among Stone, Sand and Gravel Mine Workers: Job Satisfaction as a Mediator

**DOI:** 10.3390/ijerph17061925

**Published:** 2020-03-16

**Authors:** Abdulrazak O. Balogun, Stephanie A. Andel, Todd D. Smith

**Affiliations:** 1 Department of Environmental and Occupational Health, Indiana University School of Public Health Bloomington, Bloomington, IN 47405, USA; abalogun@indiana.edu; 2 Department of Psychology, Indiana University Purdue University Indianapolis, Indianapolis, IN 46202, USA; sandel@iu.edu; 3 Department of Applied Health Science, Indiana University School of Public Health Bloomington, Bloomington, IN 47405, USA

**Keywords:** safety climate, job satisfaction, turnover intention, mine safety

## Abstract

Employee turnover has been linked to negative business performance outcomes, increased costs, and disruptions to operations. Research to explore predictors of turnover intention is important to the mining industry, including the stone, sand, and gravel mining (SSGM) industry. Safety climate has been linked to job satisfaction and reductions in turnover intention in other fields, but investigation within SSGM has virtually been non-existent, creating a knowledge gap. This research seeks to address this dearth of information. Cross-sectional data from 452 workers in the SSGM industry were analyzed to assess the influence of safety climate on turnover intention through job satisfaction. Mediation analyses showed that job satisfaction significantly mediated the relationship between safety climate and turnover intention. The implications of these novel findings are important for SSGM administrators. It suggests that bolstering safety programs and increasing safety climate perceptions will help increase job satisfaction and reduce turnover intention among workers in the SSGM industry.

## 1. Introduction

Employee turnover has been studied thoroughly in general industry and has been linked to negative business performance outcomes, increased training, and recruitment costs associated with replacing lost employees, disruption of operations, and other factors [1,2,3,4,5,6,7]. Antecedents to employee turnover intention have also been extensively studied. Findings indicate that job satisfaction is generally associated with decreased turnover intention [8,9], including within studies that examine these relationships from worker health [10,11] and safety perspectives [12,13,14]. Of particular interest lately is the influence of safety climate on job satisfaction and turnover intention. Research in this area is fairly limited [12,13,14], with no identifiable studies addressing all these factors within the mining industry. 

Safety climate can be described as employee perceptions related to the overall importance and prioritization of workplace safety. Positive perceptions of safety climate have been linked to trust, improved employee safety behavior outcomes, safety performance, and reduced workplace injury and illness [15,16,17,18,19,20]. Overall, safety climate is an important workplace metric, but one that has mostly been evaluated to examine associations with safety behaviors, injury rates, and other safety-related outcomes rather than business performance or organizational outcomes, including job satisfaction and turnover intention [14]. Improvements in the health and safety of employees have also been identified as a key focal point for sustainable mining operations [21] and improving business performance [22].

More research is needed to illustrate how safety influences more specific employee-centered business and organizational outcomes, including job satisfaction and turnover intention, particularly within the mining industry. This is especially the case in the stone, sand, and gravel mining (SSGM) industry. There is no easily identifiable research specifically examining the relationship between safety climate, job satisfaction, and turnover intention among SSGM workers. Amponsah-Tawiah and colleagues [23] found that poor safety leadership was positively associated with turnover intention among mine workers in Ghana. However, these miners did not work in SSGM operations (they worked in manganese and gold mines), and neither safety climate perceptions nor job satisfaction levels were assessed. Still, aspects of occupational safety and health programs were assessed, providing some initial insight into the relationship between safety perceptions and turnover intention among miners. 

The present study is conducted to examine the relationships between safety climate, job satisfaction, and turnover intention among SSGM workers in the United States. There are approximately 102,000 workers employed in more than 10,000 SSGM operations in the United States [24]. Thus, this is an important issue for numerous mining organizations. 

The present study is based on principles of social exchange theory [25] and is based on prior research findings in industries outside of mining that examined safety climate, job satisfaction, and turnover intention relationships [12,13,14]. Social exchange theory [25] provides a framework to explain the relationship between safety climate and organizational outcomes. This theory suggests employee commitment is influenced by management and the employee’s organization [26,27]. Further, this theory suggests that as one acts in ways to benefit another, an implicit obligation of reciprocity is created [28], which results in the desired outcome or performance. Thus, it is a tenable argument that employees who perceive that their organization shows concern for and makes efforts to promote safety may be more satisfied with their job and less willing to leave or seek other employment [12]. 

Based on this premise and in the context of prior research in industries outside of mining that examined safety climate, job satisfaction, and turnover intention, we posit safety climate will be positively related to job satisfaction and negatively related to turnover intention. Further, we posit that job satisfaction will be negatively associated with turnover intention among SSGM workers. Overall, we suggest perceptions of a strong safety climate will signal to employees that they are valued members of the organization, which in turn should be related to high levels of job satisfaction and fewer intentions to turnover. 

In alignment with this notion, McCaughey and colleagues [13] noted that a positive safety climate was positively associated with job satisfaction and negatively associated with turnover intention. Huang and colleagues [12] found that a negative safety climate and low job satisfaction were significantly related to turnover intention among a sample of truck drivers. More specifically, they found that job satisfaction mediated the association between safety climate (both at the organization and group level) and turnover intention among truck drivers [12]. Similarly, Smith [14] noted that both safety climate and job satisfaction were correlated with turnover intention and determined job satisfaction partially mediated the relationship between safety climate and turnover intention in a broad sample of workers across the United States. Thus, it appears that job satisfaction will either fully or partially mediate the effects of safety climate on turnover intention among SSGM workers in the present study. This relationship will be assessed in the present study. It is posited, given our model and its parameters, that the relationship between safety climate and turnover intention will be mediated by job satisfaction, which is a more proximal antecedent to turnover intention.

## 2. Materials and Methods 

### 2.1. Participants

Cross-sectional data were collected from 459 full-time workers employed in the SSGM industry. Companies included were small to medium-sized businesses. Worker categories included office, administration and professional (n = 71), laborers and equipment operators (n = 125), moving/rubber tire equipment/vehicle operators (n = 82), maintenance/mechanics (n = 83), supervisors (n = 51), miscellaneous/others (n = 5), and not identified (n = 42). Data were collected from participants completing the U.S. Mine Safety and Health Administration (MSHA) annual refresher training for their employer or at a training facility in the Midwestern United States. Participation in the anonymous survey was voluntary, and consent was obtained prior to participation. Institutional Review Board approval, as exempt status (IRB#1902635452), was granted by Indiana University, Bloomington, for this study. 

### 2.2. Measures

Safety climate was assessed using a six-item scale developed and validated by Hahn and Murphy [29]. Questions included (i) “In my workplace, workers learn quickly that they are expected to follow good safety practices”, (ii) “In my workplace, workers are told when they do not follow good safety practices”, (iii) “Where I work, workers, supervisors and management work together to ensure the safest possible working condition”, (iv) ”In my workplace, there are no significant compromises or shortcuts taken when worker health and safety are at stake”, (v) “The health and safety of workers is a high priority with management where I work”, and (vi) “I feel free to report problems or violations where I work”. All questions were assessed on a 5-point Likert scale ranging from strongly disagree to strongly agree. Cronbach’s alpha was 0.87.

Two questions were used to assess job satisfaction. They included “Generally speaking, I am satisfied with my job”, and “I am generally satisfied with the kind of work I do in this job”. Cronbach’s alpha was 0.85. Turnover intention was measured by the single item, “I frequently think of quitting my job”. These measures were adapted from previously developed instruments [30,31]. All questions were assessed on the same 5-point Likert scale ranging from strongly disagree to strongly agree.

### 2.3. Data Analysis

Following data cleaning to exclude subjects with missing or invalid responses (n = 7), data from 452 participants were included in the analysis. Descriptive statistics delineating mean and standard deviation values are presented in Table 1 for each of the study measures according to job category. A series of one-way analyses of variance (ANOVAs) were conducted to examine if perceptions of safety climate, job satisfaction, and turnover intention were significantly different across job categories. 

Next, Pearson’s correlation coefficients were used with the full sample to assess the relationships between all variables. The correlation matrix is presented in Table 2, along with the mean and standard deviation values for the full sample measures. Finally, Hayes’ PROCESS macro (Model 4) for SPSS [32] was used with the full sample to estimate the proposed indirect effect. The indirect effect was interpreted using bias-corrected 95% confidence intervals (CIs) with 5000 bootstrapped iterations. All analyses were conducted using SPSS Version 25.

## 3. Results

The one-way ANOVA results showed that perceptions of safety climate (*F*(6, 446) = 1.375, *p* = 0.223), job satisfaction (*F*(6, 448) = 0.649, *p* = 0.691), and turnover intention (*F*(6, 449) = 1.103, *p* = 0.360) did not significantly differ across job categories (see Table 1 for descriptive statistics of the study variables broken down by job category). Therefore, the correlation coefficients were calculated using the full sample. Descriptive statistics and correlations are presented in Table 2. 

As is evident in the correlation matrix, safety climate was significantly, positively correlated with job satisfaction (*r* = 0.478, *p* < 0.001). Further, safety climate was significantly, negatively correlated with turnover intention (*r* = −0.326, *p* < 0.001). A significant, negative correlation was also found between job satisfaction and turnover intention (*r* = −0.515, *p* < 0.001).

**Figure 1:**
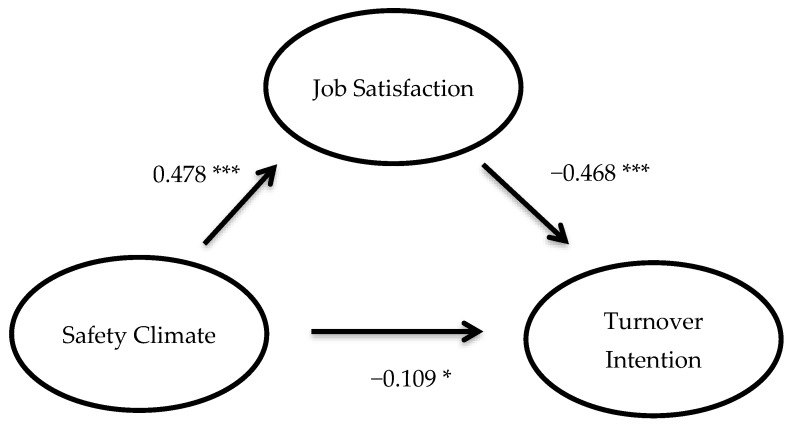
Path diagram illustrating the mediating role of job satisfaction on the association between safety climate and turnover intention. ^*^ *p* < 0.05; ^***^ *p* < 0.001.

Results of the mediation analysis with the full sample showed that safety climate was a significant predictor of job satisfaction (β = 0.478, *p* < 0.001, *R^2^* = 0.228). Further, job satisfaction was a significant predictor of turnover intention (β = −0.468, *p* < 0.001) and the direct effect of safety climate on turnover intention (while controlling for job satisfaction) was also significant (β = −0.109., *p* < 0.05, *R^2^* = 0.279). Finally, the indirect effect of safety climate on turnover intention through job satisfaction was significant (unstandardized indirect effect = −0.382, 95% CIs = −0.502, −0.274; Figure 1). Thus, our main hypothesis is supported. 

Follow-up analyses were also conducted to see if the study results remained consistent after removing office workers (n = 71) from the sample. The results of the mediation analysis remained largely the same. Safety climate was a significant predictor of job satisfaction (β = 0.462, *p* < 0.001, *R^2^* = 0.214). Further, job satisfaction was a significant predictor of turnover intention (β = −0.464, *p* < 0.001), and the direct effect of safety climate on turnover intention (while controlling for job satisfaction) was not significant (β = −0.093., *p* = 0.063, *R^2^* = 0.264). The indirect effect of safety climate on turnover intention through job satisfaction was significant (unstandardized indirect effect = −0.368, 95% CIs = −0.502, −0.250). Overall, evidence suggests the study results are quite robust, regardless of whether office workers are included in the analyses or not.

## 4. Discussion

This study sought to examine relationships between safety climate, job satisfaction, and turnover intention among employees working in the SSGM industry. There are approximately 102,000 employees working in this industry across more than 10,000 SSGM operations [24]. Maintaining a qualified and experienced workforce is important to these SSGM mine operators, particularly since turnover impacts productivity, performance, and expenses [1,3,5,6,7], and since there are approximately 1500 non-fatal lost-time injuries within SSGM operations on an annual basis [24].

A focus on safety by mine administrators can be extremely beneficial not only with protecting workers, property, the efficiency of operations, and productivity but also with enhancing safety climate perceptions, which, based on the findings of this study, impact job satisfaction and turnover intention among mine workers. The findings from this study illustrate how safety climate and job satisfaction are associated with turnover intention. Particularly, job satisfaction plays a mediating role between safety climate and turnover intention. This is a novel finding within SSGM operations, though not completely surprising. Huang et al. [12] found that job satisfaction mediated the association between safety climate (both at the organization and group level) and turnover intention among truck drivers, and Smith [14] determined that job satisfaction partially mediated the relationship between safety climate and turnover intention in a broad sample of workers across the United States. Both Huang et al. [12] and Smith [14] suggest these relationships are attributed to reciprocity in the context of social exchange theory. In this context, workers are obligated to respond in kind when treated favorably by the employer [12]. Thus, they are less likely to leave the organization to pursue employment outside the company [14]. In addition to reducing turnover, Huang et al. suggest that a positive safety climate may also increase other human resource outcomes through the same social exchange process [12].

The results of this study underscore the importance of a safety climate in the SSGM industry. It is evident that positive safety climate perceptions influence job satisfaction and employee/talent retention. Thus, mine administrators should focus on bolstering safety climate perceptions among workers. Evidence suggests that safety climate perceptions are attributed to factors such as management commitment to safety, enactment of safety programs, policies, and procedures, supervisor support for safety, open communication, and safety systems, among other factors [16,20,33,34,35]. 

Although definitions and dimensions of safety climate vary somewhat within the literature, management commitment to safety has consistently been one of the strongest indicators of a positive safety climate [14,33,34,36]. As such, management within SSGM operations should not only espouse that safety is important, but should demonstrate that safety is paramount and is, in fact, a major value within the organization. In a highly regulated industry such as mining, some administrators have traditionally thought that regulatory compliance is indicative of a strong safety program. Although compliance with regulations is important and essential in the mining industry, this is the minimum that should be done to protect workers. If organizations simply focus on compliance and control-based safety, they will not likely get mine workers to reciprocate and perform at their safest and best, particularly in comparison to commitment-based safety approaches [37]. Consequently, in order to attain high levels of safety performance, top administrators and management must “perform” in the context of safety. They must take action, illustrate commitment, and not simply “talk” of the importance of safety. 

Empirical studies have shown that safety climate can also be meaningfully influenced through other specific ways, such as by training supervisors to prioritize safety issues in daily communications with subordinates [38], teaching supervisors to engage in safety-specific leadership behaviors (i.e., promoting safety across levels of the organization through individualized consideration for employees, inspirational motivation, idealized influence, and intellectual stimulation); [39], and promoting continuous feedback and peer feedback among employees [40].

Attractively, management commitment to safety and positive safety climate perceptions not only enhance job satisfaction and turnover intention, but both also influence other factors important to operational productivity and success. Safety climate has been linked to increased levels of trust [19], work engagement and improved organizational performance [41], enhanced quality of work life, including within the mining industry [42], and controlling job demands [43,44]. These results lend support for safety climate to be used as a leading indicator for employee health and safety and business performance.

In addition to focusing on safety climate, our results also show that job satisfaction is another (and more proximal) indicator of turnover intention. Accordingly, it would also be beneficial for organizations in the SSGM industry to attend to employee satisfaction levels in order to reduce turnover intention among employees. For instance, research suggests that efforts to increase opportunities for employees to provide feedback about organizational processes [45] and training supervisors in transformational leadership behaviors [46] are two strategies for effectively increasing employee job satisfaction.

Although the findings associated with this study are novel and have implications for the SSGM industry, some limitations exist and must be considered when interpreting the results of the study. Safety climate in the present study was examined at the individual level since participants were part of a convenience sample and were employed by multiple companies. It would be beneficial to complete analyses within larger mining companies to determine if these same relationships were evident, particularly in the context of shared perceptions of safety climate at the workgroup and organizational level. Future research within the SSGM industry might incorporate these strategies assessing safety climate. Further, more tailored safety climate measures could be incorporated. Although the safety climate scale [29] used in this study has been validated and its reliability was good, it would be beneficial to examine whether more targeted measures specific to safety climate in SSGM at the workgroup and organizational level might be associated with job satisfaction and turnover intention. This would obviously be a large undertaking, as it would require substantive efforts to develop and refine measures relevant to SSGM operations. Additionally, the present study included cross-sectional data. This limits our ability to claim causality. Longitudinal studies assessing the influence of safety climate on job satisfaction and turnover intention over time would be a welcome addition to the literature, particularly if the researcher could extract how these predictors influence turnover intention beyond other contributing or confounding factors. In addition, additional qualitative approaches could be applied in future research. Interviews with mine workers could also be conducted in future studies to further examine the relationships explored in this study. These qualitative approaches might provide additional insights regarding the relationships between the variables in our study without the constraints of survey research. Lastly, this study was conducted in the Midwestern United States. Thus, our findings may not be fully generalizable to all workers in the aggregates or SSGM industry. Future research should aim to include a broader sample of workers across the United States and even internationally.

## Figures and Tables

**Table 1:** Descriptive statistics by job category.

	Safety Climate		Job Satisfaction		Turnover Intention	
Job Category	*M*	*SD*	*M*	*SD*	*M*	*SD*
Office/Administrative/ Professional	4.120	0.721	4.127	0.917	2.028	1.206
Laborers/Equipment Operators	3.992	0.746	4.198	0.695	2.226	1.202
Moving/Rubber Tire Equipment/Vehicle Operators	4.008	0.706	4.253	0.716	2.210	1.180
Maintenance/Mechanics	3.868	0.678	4.183	0.743	2.293	1.171
Supervisors	4.142	0.678	4.320	0.873	2.294	1.331
Miscellaneous/Others	4.033	0.558	4.300	0.447	2.000	0.707
Job Category Missing	3.862	0.800	4.048	0.854	2.619	1.431

**Table 2:** Descriptive statistics and correlation matrix with full sample.

Measure.	Items	*M*	*SD*	Safety Climate	Job Satisfaction	Turnover Intention
Safety Climate	6	3.998	0.721	1.000		
Job Satisfaction	2	4.195	0.778	0.478 ***	1.000	
Turnover Intention	1	2.246	1.228	−0.326 ***	−0.515 ***	1.000

^***^p < 0.001
